# Postabortion care availability, readiness, and accessibility in Niger in 2022: results from linked facility-female cross-sectional data

**DOI:** 10.1186/s12913-023-10107-4

**Published:** 2023-10-27

**Authors:** Haley L. Thomas, Souleymane Alzouma, Sani Oumarou, Caroline Moreau, Suzanne O. Bell

**Affiliations:** 1 Department of Population, Family and Reproductive Health, Johns Hopkins University Bloomberg School of Public Health, 615 N. Wolfe Street., Baltimore, MD 21205 USA; 2 Institut National de la Statistique du Niger, Niamey, Niger; 3 Soins Primaires et Prévention, CESP Centre for Research in Epidemiology and Population Health, U1018, Inserm, Villejuif, F-94800 France

**Keywords:** Postabortion care, Abortion, Niger, Survey, Health facilities

## Abstract

**Background:**

Postabortion care (PAC), which is an essential element of emergency obstetric care, is underresearched in Niger. The study aims to assess the availability, readiness, and accessibility of facility-based PAC services in Niger.

**Methods:**

This study uses female and facility data from Performance Monitoring for Action Niger. The female data include a nationally representative sample of women aged 15–49 (n = 3,696). Using GPS coordinates, these female data were linked to a sample of public and private facilities (n = 258) that are expected to provide PAC. We assessed PAC availability and facility readiness to provide basic and comprehensive PAC using the signal functions framework, overall and by facility type. We then calculated the distance between women and their closest facility and estimated the proportion of women living within five kilometers (5 km) of a facility providing any PAC, basic PAC, and comprehensive PAC, overall and by women’s background characteristics.

**Results:**

Only 36.4% and 14% of eligible facilities had all basic and comprehensive PAC signal functions, respectively. Oxytocics and laparotomy were the most missing signal function for basic and comprehensive PAC, respectively. Private facilities were the least ready to provide the full range of PAC services. While 47% of women lived within 5 km of a facility providing any PAC services, only 33.4% and 7.9% lived within 5 km of a facility providing all basic and all comprehensive PAC signal functions, respectively. Women who were divorced/widowed, had higher levels of education, and were living in urban areas had increased odds of living within 5 km of a facility with any or basic PAC. Women who were never married had increased odds of living within 5 km of a facility with comprehensive PAC, while urban residence was fully predictive of living within 5 km of a facility with comprehensive PAC.

**Conclusions:**

This study found PAC availability and readiness to be insufficient in Niger, with inadequate and disparate accessibility to facilities providing PAC services. We recommended stakeholders ensure stock of essential commodities and availability of PAC services at primary facilities in order to mitigate the negative maternal health repercussions of unsafe abortion in this setting.

## Background

Despite induced abortion being one of the safest procedures when performed according to recommended guidelines, unsafe induced abortion is a leading contributor to maternal mortality worldwide [1, 2]. Nearly half of all abortions globally (45.1%) are considered unsafe [3], characterized as being performed by either untrained individuals and/or with non-recommended methods [2]. Unsafe abortion is responsible for 7.9% (4.7–13.2%) of all maternal deaths annually [4] and is most prevalent in low-resource countries and countries with restrictive abortion laws [3]. In sub-Saharan Africa (SSA), 77% of all abortions are unsafe, and the region has the highest unsafe abortion case fatality rate in the world (185 deaths per 100,000 abortions), leading to 15,000 preventable deaths annually [5]. In contexts where restrictive abortion laws prevent access to safe abortion methods and providers, quality postabortion care (PAC), which includes a constellation of services for treating abortion complications [6], is necessary to mitigate the negative sequelae of unsafe abortion such as maternal morbidity and mortality.

Most countries in SSA have pledged to provide or expand PAC to ensure that women have access to formal health services that treat induced abortion complications regardless of their country’s legal grounds for abortion [7]. This is critical for improvement in maternal health outcomes broadly given PAC services are also needed to treat complications from spontanous abortion (miscarriage) and childbirth. Since making these pledges at the International Conference for Population and Development in 1994, many countries have expanded PAC services, yet the extent and quality of these services remains unknown in a number of SSA countries [8].

Facilities’ ability to provide quality PAC services depends on their *readiness* to provide all components, or signal functions—adapted from the United Nations’ emergency obstetric care (EmOC) signal functions [9]—to provide safe abortion care (SAC), including services following an abortion. SAC signal functions include a range of services, equipment, and commodities, such as removal of retained products of conception, administration of antibiotics, oxytocics, and intravenous (IV) fluids, and provision of postabortion contraception, among others [10]. Facilities are considered *ready* to provide quality PAC services if they have all signal functions for basic and comprehensive PAC. Basic PAC includes the ability to treat minor complications and provide services at early gestations (≤ 12 weeks) while comprehensive PAC includes the ability to treat more severe complications and provide services at later gestations (> 12 weeks) [10]. Investigators have used this framework to assess PAC services in a number of SSA countries recently [11–15], with results generally suggesting inadequate service availability and readiness. Additionally, one study in Nigeria and Côte d’Ivoire linked facility-based data to contemporaneously collected population-based data to show significant disparities in access to facilities offering basic and comprehensive PAC services [15].

Niger, where abortions are only permitted in cases of fetal impairment or to save a woman’s life or health [16], has a dearth of information on abortion, including PAC services. In 2021, abortion incidence was estimated at 6.7 abortions per 1,000 women of reproductive age, equivalent to 36,856 annual induced abortions [17]. More than 90% of these were reported as unsafe, and 43% of women reported experiencing a potentially severe complication (e.g., fever, vaginal discharge, or complications requiring surgery); however, less than half sought PAC from a facility [17]. This may be due to fear of social or legal repercussions, a lack of service availability, uncertainty regarding the quality of the services available, or an inability to access PAC services.

PAC services were first introduced as a “lifesaving safe motherhood intervention” in Niamey, Niger in 2001 when it was estimated that nearly 500 women were dying each year from unsafe abortion [18]. Though PAC services have expanded throughout the country since then, progress has likely been limited in part due to high turnover within the Ministry of Health, resulting in logistical and financial barriers to PAC implementation [16]. Niger’s health care is primarily provided through the public health care system, which has three tiers—national (including national and maternity hospitals), regional (including regional hospitals and mother and child health centers), and peripheral (including district hospitals, integrated health centers, and health huts), with referral facilities at the national and regional tiers and primary facilities at the peripheral tier [19]. Though we do not know the extent of basic and comprehensive PAC service availability at different levels of the health care system in Niger, we know the availability and accessibility of health care services in general is inadequate; the overall numbers of doctors and nurses per capita are below WHO standards [19], and most healthcare staff (76%) are located in urban areas despite 80–90% of the population living in rural localities [19], resulting in more than half of the population having to travel more than five kilometers to access basic health services [20].

Further work is needed to understand PAC availability and accessibility in Niger. The study addresses this gap by (1) assessing PAC availability and facility readiness to provide PAC services and (2) examining disparities in the accessibility of facilities providing PAC.

## Methods

### Data and samples

Data for this study come from Performance Monitoring for Action (PMA), a research platform that administers representative female surveys and service delivery point (SDP) surveys serving the same geographies as the female surveys in eight countries in sub-Saharan Africa and Asia to monitor family planning and women’s health indicators. Surveys are administered by trained female resident interviewers using smartphones with the Open Data Kit (ODK) software.

#### Female sample

In Niger, PMA used a multi-stage stratified cluster design with probability proportional to size sampling of 103 geographic areas to obtain a nationally representative sample of 3,515 households (98.8% response rate) including 3,633 females aged 15–49 (95.4% response rate). Geographic and household selection occurred between December 2020 and April 2021. For this analysis we used Phase 2 female data that were collected between January and May of 2022. The sample followed households and women from the first round of data collection as well as a random sample of replacement households in geographic clusters with more than 10% loss to follow-up. The final Phase 2 sample included 3,696 females ages 15–49 (96.3% response rate). Surveys were administered face-to-face with participating women in French or local language, and responses were recorded on smartphones via the ODK platform.

#### SDP sample

All public facilities that served the female survey geographic clusters, including primary and referral facilities, and up to three private facilities within each geographic cluster were invited to participate in the SDP survey; if there were more than three private SDPs per cluster, three were randomly selected. In total 288 facilities (response rate 97.6%) participated, from which 258 were selected based on their potential for providing postabortion care; this excluded pharmacies and shops. These facilities serve the nationally representative sample of women. SDP survey respondents were facility management staff and/or providers, depending on who was most knowledgeable about the content of the survey; this could also vary by survey section.

All female respondents provided verbal informed consent to participate; no informed consent was required for the facility survey. The Johns Hopkins University Bloomberg School of Public Health institutional review board and the Comité Consultatif National d’Ethique in Niger provided ethical approval for the study protocol. Additional details about the PMA project can be found at pmadata.org.

### Measures

The SDP sample included public and private facilities at all levels of the healthcare system. We distinguished facility types by their level and sector, generating three categories for analysis - public referral, public primary, and private. We grouped all publicly managed hospitals and Center Madonna’s (mother and child health centers) as public referral facilities. Integrated centers and health huts were grouped as public primary facilities, and polyclinics (which provide specialized care and/or surgery), private rooms and private practices, as well as other facilities that were privately managed, were grouped together as private facilities. All three facility types should have the capacity to provide basic PAC, and public referral should have the capacity to provide comprehensive PAC. While some private facilities have the capacity to provide comprehensive PAC, we were unable to distinguish between primary and referral level private facilities due to the smaller number of private facilities in Niger and thus in our sample of facilities.

To capture facilities’ ability to provide PAC services, those included in the SDP sample were asked, “Which of the following services are provided at this facility?” Basic PAC signal functions include ≤ 12 weeks’ removal of retained products of conception, antibiotics, oxytocics, intravenous replacement fluids and provision of any contraceptive methods (Table 1). Comprehensive PAC signal functions include all basic PAC signal functions plus > 12 weeks’ removal of retained products of conception, blood transfusion, laparotomy, 24/7 PAC services availability, and provision of long-acting reversible contraception (LARC). Medication-related signal functions were considered available if they were in-stock and observed on the day of the survey.

**Table 1:** Basic and comprehensive PAC signal functions criteria

Basic PAC signal Functions ≤12 weeks’ removal of retained products of conception Antibiotics Oxytocics Intravenous replacement fluids Any contraception Comprehensive PAC Signal Functions All basic PAC signal functions >12 weeks’ removal of retained products of conception Blood transfusion Laparotomy 24/7 PAC services available Long-acting reversible contraception	

In the female sample, we identified sociodemographic chatacteristics to examine disparities in access to PAC services. Characteristics included age (15–19, 20–29, 30–39, 40–49), education (none, primary, secondary or higher), marital status (currently married/cohabiting, divorced/widowed, never married), wealth (poorest, middle wealth, wealthiest, generated via principal component analysis based on household assets, building materials, and water and sanitation sources), residence (urban, rural), and region (Niamey, other).

### Analysis

We first examined the distribution of facilities in Niger by facility type and region. We then estimated the proportion of facilities providing each of the signal functions, overall and by facility type. Next, we estimated the proportion of facilities with all basic and comprehensive PAC signal functions overall and by facility type and region. Lastly, we generated a readiness score for basic and comprehensive PAC for each facility, which we calculated as the percentage (out of 100%) of signal functions available; for basic PAC this was out of five signal functions and for comprehensive PAC this was out of 10 signal functions. We averaged the scores to produce readiness scores overall and by facility type and region.

To examine disparities in the geographic accessibility of PAC services as measured by proximity, we linked facility data to the representative sample of women aged 15–49 using geospatial information. We estimated the proportion of women living within 5 km of a facility that provided any PAC, a facility with all basic PAC signal functions, and a facility with all comprehensive PAC signal functions, overall and by women’s sociodemogrpahic characteristics. We then ran bivariable and multivariable logistic regressions to examine the association between sociodemographic characteristics and within-5 km-access to any, basic and comprehensive PAC. All analyses were conducted in Stata version 16, and analyses involving female data were weighted to account for the complex survey design, including probability of selection and non-response, and adjusted for clustering.

## Results

### Facility characteristics

The majority of the 258 health facilities that should have the capacity to provide PAC were public primary facilities (64%), followed by public referral facilities (18.6%) and private facilities (17.4%) (Table 2). The distribution of health facilities varied across the country with greatest concentration in the capital city of Niamey (39.9%), where 8.8% of the population of reproductive-aged women resides (population distribution not presented in Table 2).

**Table 2:** Health facility characteristics of facilities in Niger

	n	%
Facility Type		
Public Primary	165	64.0
Public Referral	48	18.6
Private	45	17.4
Region		
Agadez	13	5.0
Diffa	10	3.9
Dosso	26	10.1
Maradi	30	11.6
Niamey	103	39.9
Tahoua	27	10.5
Tillaberi	22	8.5
Zinder	27	10.5
Total	258	100.0

### PAC signal functions and facility readiness to provide PAC services

Overall, 62.4% of the 258 facilities provided removal of retained products of conception for pregnancies ≤ 12 weeks’ gestation, and 53.1% provided this service for pregnancies > 12 weeks’ gestation (Table 3). The most commonly available signal functions across facility types were antibiotics and LARCs for basic and comprehensive PAC, respectively. Oxytocics and IV fluids were the basic PAC signal functions most commonly unavailable, while laparotomy was the comprehensive PAC signal function most often unavailable.

**Table 3:** Health facilities with selected basic and comprehensive PAC signal function indicators, overall and by facility type in Niger

	Public Primary		Public Referral		Private		Overall	
	*n*	%	*n*	%	*n*	%	*n*	%
BASIC PAC SIGNAL FUNCTIONS								
≤ 12 weeks’ removal of retained products of conception	113	68.5	37	77.1	11	24.4	161	62.4
Antibiotics	157	95.2	47	97.9	41	91.1	245	95.0
Oxytocics	106	64.2	35	72.9	11	24.4	152	58.9
Intravenous replacement fluids	96	58.2	43	89.6	21	46.7	160	62.0
Any contraception	163	98.8	39	81.3	15	33.3	217	84.1
COMPREHENSIVE PAC SIGNAL FUNCTIONS								
> 12 weeks’ removal of retained products of conception	90	54.6	36	75.0	11	24.4	137	53.1
Blood transfusion	8	4.9	40	83.3	12	26.7	60	23.3
Laparotomy	2	1.2	24	50.0	5	11.1	31	12.0
24/7 PAC services available	97	58.8	34	70.8	7	15.6	138	53.5
Long-acting reversible contraception	149	90.3	38	79.2	13	28.9	200	77.5
Total	165	100.0	48	100.0	45	100.0	258	100.0

Only 36.4% of facilities that had the capacity to provide any PAC had all basic PAC signal functions (Table 4). A higher proportion of public referral facilities had all basic PAC signal functions compared to public primary and private facilities (64.6% compared to 35.8% and 8.9%, respectively). The proportion of facilities with all basic PAC signal functions was highest in Niamey (20.2%) and Tillaberi (20.2%) and lowest in Diffa (2.1%). Among facilities with the capacity to provide comprehensive PAC, only 14% had all corresponding signal functions. A quarter of public referral facilities had all comprehensive PAC signal functions while only 2.2% of private facilities met this criterion. Regionally, the proportion of facilities with all comprehensive PAC signal functions ranged from 23.1% in Dosso and Tahoua to 0% in Tillaberi.

**Table 4:** Percentage of facilities that have all basic and comprehensive PAC signal function indicators and the readiness score overall and by facility type and region in Niger

	Basic PAC			Comprehensive PAC		
	*n*	% with all components	Readiness score*	*n*	% with all components	Readiness score*
Overall	*258*	36.4	72.5	*93*	14.0	55.9
Facility Type						
Public Primary	165	35.8	77.0	--	--	--
Public Referral	48	64.6	83.8	48	25.0	77.7
Private	45	8.9	44.0	45	2.2	32.7
Region						
Agadez	13	5.3	80.0	3	7.7	66.7
Diffa	10	2.1	74.0	3	7.7	70.0
Dosso	26	10.6	73.1	9	23.1	65.6
Maradi	30	9.6	75.3	10	15.4	70.0
Niamey	103	20.2	60.2	43	7.7	37.4
Tahoua	27	16.0	80.0	11	23.1	67.3
Tillaberi	22	20.2	92.7	8	0.0	78.8
Zinder	27	16.0	87.4	6	15.4	86.7

Despite few facilities having all basic and comprehensive PAC signal functions, many had the majority of them, as indicated by the readiness scores (Table 4). Facilities with the capacity to provide basic and comprehensive PAC had on average 72.5% and 55.9% of the corresponding signal functions, respectively. Public referral facilities had the highest readiness scores for both basic and comprehensive PAC. There was substantial variation in readiness score by region, with facilities in Niamey having the lowest scores for basic and comprehensive PAC.

### Access to PAC services among women of reproductive age

Most women did not live in proximity to a factility providing PAC services. Overall, 47%, 33.4% and 7.9% of women aged 15–49 lived within 5 km of a facility providing any PAC, basic PAC, or comprehensive PAC, respectively. Access to PAC services varied by women’s sociodemographic characteristics, with decreased access among poorer, less educated, married/cohabiting, and rural women (Table 5). There was also substantial variability in access by region based on PAC type. In Agadez, 100% of women lived within 5 km of a facility that provided any PAC, but 0% of women lived within 5 km of a facility that had all comprehensive PAC signal functions. Similarly, the majority of women in Niamey lived within 5 km of a facility that provided any and all basic PAC signal functions (97.4% and 95%, respectively), but only 39.2% lived within 5 km of a facility that provided comprehensive PAC.

**Table 5:** Percent of women aged 15–49 living within five kilometers of a facility providing any PAC and those with all basic and comprehensive PAC signal functions by background characteristics in Niger

	*n*	Any PAC	Basic PAC	Comprehensive PAC
		%*	%*	%*
Overall	3696	47.0	33.4	7.9
Age				
15–19	821	52.0	37.5	10.5
20–29	1400	48.0	33.9	7.5
30–39	932	43.3	30.5	6.8
40–49	543	44.0	31.6	7.4
Education				
None	1747	34.4	22.5	3.9
Primary	697	59.4	40.0	8.9
Secondary or higher	1251	82.8	68.9	22.7
Marital Status				
Currently married/cohabiting	2656	42.2	28.3	5.4
Divorced/widowed	193	70.7	62.2	14.9
Never married	847	69.8	56.9	21.1
Residence				
Urban	2019	97.5	93.6	42.6
Rural	1677	35.5	19.7	0.0
Wealth tertile				
Poorest	686	23.6	12.8	0.2
Middle	670	37.3	21.0	0.6
Wealthiest	2340	76.3	62.7	21.2
Region				
Agadez	87	100.0	23.7	0.0
Diffa	90	14.4	14.4	14.4
Dosso	315	36.1	35.3	8.2
Maradi	522	51.7	21.3	8.4
Niamey	1359	97.4	95.0	39.2
Tahoua	486	31.6	26.1	0.0
Tillaberi	234	50.1	50.1	0.0
Zinder	603	38.1	19.4	5.9

Compared to women with no formal education, women who had attended primary school or higher had two to three times the odds of living within 5 km of a facility that provided any PAC (aOR_Primary_=2.24, 95% CI 1.60–3.13; aOR_Secondary or Higher_=3.08, 95% CI 1.72, 5.51) (Table 6). Similarly, they had an increased odds of living within 5 km of a facility that provided basic PAC (aOR_Primary_=1.67, 95%CI 1.08–2.57; aOR_Secondary or Higher_=1.94, 95% CI 1.05, 3.59). Divorced/widowed women had greater odds of living within 5 km of facility that provided any PAC (aOR = 2.58, 95% CI 1.25–5.36) and basic PAC (aOR = 3.71, 95% CI 1.70, 8.11) when compared to currently married/cohabiting women, and never married women had an increased odds of living within 5 km of a facility providing comprehensive PAC (aOR = 1.82, 95% CI 1.22, 2.71) when compared to married/cohabiting women. Rural women had substantially decreased odds of living near a facility that provided any or basic PAC when compared to urban women (any PAC, aOR = 0.05, 95% CI 0.00, 0.54; basic PAC, aOR = 0.05, 95% CI 0.01, 0.27); urban residence was fully predictive of living within 5 km of a facility with comprehensive PAC. Household wealth was also associated with access to any PAC services, with the wealthiest women having about three times the odds of living within 5 km of a facility offering any PAC services (aOR = 3.36, 95% CI 1.40–8.07) while women in the middle wealth tertile had 1.81 (95% CI 1.05–3.12) times the odds of living within 5 km of a facility offering any PAC services when compared to the poorest women.

**Table 6:** Adjusted odds ratio of living within five kilometers of a facility providing basic PAC and comprehensive PAC among women aged 15–49 in Niger

	Any PAC aOR (95% CI)	Basic PAC aOR (95% CI)	Comprehensive PAC aOR (95% CI)
Age			
15–19	(ref)	(ref)	(ref)
20–29	**1.41 (1.00, 1.99)** ^*^	1.46 (1.00, 2.14)	1.30 (0.93, 1.81)
30–39	1.27 (0.81, 2.00)	1.23 (0.76, 1.98)	0.99 (0.63, 1.56)
40–49	1.39 (0.94, 2.05)	1.27 (0.81, 1.98)	1.11 (0.63, 1.97)
Education			
None	(ref)	(ref)	(ref)
Primary	**2.24 (1.60, 3.13)** ^***^	**1.67 (1.08, 2.57)** ^*^	1.01 (0.71, 1.43)
Secondary or higher	**3.08 (1.72, 5.51)** ^***^	**1.94 (1.05, 3.59)** ^*^	0.62 (0.36, 1.08)
Marital Status			
Currently married/cohabiting	(ref)	(ref)	(ref)
Divorced/widowed	**2.58 (1.25, 5.36)** ^*^	**3.71 (1.70, 8.11)** ^***^	1.48 (0.98, 2.25)
Never married	1.41 (0.80, 2.47)	1.46 (0.66, 3.23)	**1.82 (1.22, 2.71)** ^**^
Residence			
Urban	(ref)	(ref)	
Rural	**0.05 (0.00, 0.54)** ^*^	**0.05 (0.01, 0.27)** ^***^	^§^
Wealth tertile			
Poorest	(ref)	(ref)	(ref)
Middle	**1.81 (1.05, 3.12)** ^*^	1.67 (0.90, 3.06)	1.13 (0.09, 13.47)
Wealthiest	**3.36 (1.40, 8.07)** ^**^	2.88 (1.00, 8.31)	3.88 (0.58, 25.99)
Region			
Niamey	(ref)	(ref)	(ref)
Other	0.47 (0.03, 6.74)	0.35 (0.07, 1.67)	1.19 (0.35, 4.07)

^*^<0.05; ^**^<0.01; ^***^<0.001

^§^ Urban residence is fully predictive of whether women live with five kilometers of a facility with all comprehensive PAC signal functions

## Discussion

Our findings indicate that few facilities in Niger are equipped with all signal functions necessary to provide quality PAC services. However, based on readiness scores, we found that facilities reported, on average, half of all signal functions, allowing them to provide critical elements of PAC. Oxytocics and IV fluids were the most commonly missing basic PAC signal functions and laparotomy was the most commonly missing comprehensive PAC signal function, with oxytocic shortages and the inability to perform laparotomies being more pronounced among private facilities, which is likely a result of the fact that private facilities were more predominantly primary level. Additionally, availability and readiness of PAC services also varied by geography, ultimately resulting in social disparities in proximity to PAC services, with decreased access among poorer, less educated, married/cohabiting, and rural women.

While there are no studies that have previously explored PAC in Niger, our results are broadly consistent with findings from other SSA countries suggesting inadequate PAC service availability and quality [12, 13, 15, 21, 22]. Our findings also corrobarate a recent systematic review highlighting the lack of emergency obstetric services and supplies in SSA across all system levels (primary and advanced care), contributing to preventable maternal deaths, including unsafe abortion fatalities [23]. While health system’s deficiencies call for additional investment to improve quality of EmOC,  including PAC, they also need to be integrated into a continnum of care to improve maternal health, starting with family planning as a primary prevention of unintended pregnancy and continuing with the diffusion of safer methods to terminate pregnancies and prevent abortion complications. Our findings show high proportions of public primary and public referral facilities providing at least one method of contraception, however, private facilities were often lacking. This similar trend was seen regarding LARCs, indicating a potential inability for private facilities to help PAC-seeking women prevent future unintended pregnancies. Such insufficiencies in PAC contraceptive services at different levels of the health system have been noted elsewhere [11, 24].

Healthcare deficiencies were not equally distributed, with our results showing stark variations in PAC availability and readiness between public and private facilities, both across and within regions. These geographic deficiencies also translate to social disparities, as women in rural areas and women from disadvantaged social backgrounds were less likely to live in proximity to a functional PAC facility. As such, healthcare deficiencies, which mirror the geographic distribution of emergency obstertic care in the sub-Saharan region more generally [25], further compound social disparities in maternal health, as population based studies consistenly indicate that women from rural areas or from disadvantaged social backgrounds are less likely to use contraception to prevent an unintended pregnancy and more likely to rely on unsafe means to terminate a pregnancy when seeking an abortion. Previous studies have also indicated that the same women are less likely to seek care in case of abortion related complications, which may be due service inavailability and/or anticipated poor treatment [26].

This study has several strengths. To our knowledge, it is the first study to estimate and describe PAC in Niger. Using PMA SDP data, we were not only able to assess overall availability of PAC services, but we were also able to estimate facilities’ readiness to provide the full range of PAC services. We also generated a readiness score, which provides more nuance in quantifying readiness, as some facilities may be close to “ready”. Additionally, by looking at the availability of individual signal functions, we were able to determine facilities’ limiting PAC signal functions, overall and by facility type, indicating specific areas for improving PAC services at different levels of the healthcare system. Lastly, by leveraging contemporaneously collected female and facility data, we were able to link the sample to assess disparities in PAC access. Inaccessibility of PAC services has the potential to lead to disparities in reproductive health outcomes, such as severe morbidity and mortality. By identifying vulnerable women, we can target specific areas of intervention to mitigate preventable, unsafe abortion-related harms.

This study is not without limitations. First, while the sample of facilities represents the service delivery environment for a nationally representative sample of reproductive-aged women in Niger, the facilities may not be representative of all facilities in the country as we were not able to obtain a recent national sampling frame of public and private facilities from which to construct facility weights. Thus, results should be interpreted accordingly. We were unable to stratify between private primary and private referral facilities due to the small sample of private facilities, which may differ in their ability to provide PAC services. Our measure of PAC readiness did not include indicators for recent provider training, the availability of PAC guidelines, or facilities’ referral capacity, which have been used in other PAC readiness studies [24, 27, 28].While we chose to focus on indicators we thought were most critical to the delivery of PAC, the absence of these additional indicators in our assessment may mean our readiness assessments are overestimates. While not an aim of the study, our measure of PAC readiness also does not allow us to determine the quality of these services more broadly. Finally, we operationalized accessibility to PAC services as geographic proximity; however, there are other barriers to accessibility that may prevent women from seeking care even if they are geographically close, such as costs, transportation, knowledge of services, and fear of social stigma and sanctions [29].

Despite these limitations, our findings have important implications for in-country stakeholders as they work to expand PAC services, starting with basic PAC readiness at the primary care setting and expanding to higher level care for more serious complications. Given facilities’ lack of oxytocics and IV fluids overall, and the even more severe lack of oxytocics, IV fluids and contraceptives at private facilities, we recommend a supply chain evaluation to determine the barriers to stocking these critical PAC commodities at all facility levels across the healthcare system. Overcoming stock barriers could increase the number of facilities that are able to provide the full range of PAC services. Additionally, given disparities in access to facilities providing PAC services, we emphasize the importance of understanding barriers to healthcare utilization among disadvantaged groups, as those groups may experience compounding impacts from unintended pregnancy, unsafe abortion, abortion complications, and insufficient PAC. We also emphasize the need for improved primary care services more broadly given the majority of women, particularly rural and poor women who are less likely to have access to PAC, access health care. Finally, expanded EmOC to serve these populations would improve maternal health outcomes beyond induced abortion such as spontaneous abortion, which results in about 15% of all recognized pregnancies [30], though not all would result in complications requiring EmOC.

## Conclusions

This study found PAC availability and readiness to be insufficient in Niger, with social inequalities in accessibility to facilities providing PAC services was not equal for all women across sociodemographic groups. Our findings highlight specific aspects of PAC that can be targeted to improve service provision in Niger. We recommended that stakeholders focus on stocking essential commodities and exploring ways to mitigate barriers to PAC accessibility in order to mitigate the negative maternal health repercussions of unsafe abortion in this setting.

## Data Availability

PMA data are publicly available and can be accessed by submitting a request through the PMA website: https://datalab.pmadata.org/.

## Abbreviations

SSA: Sub-Saharan Africa
PAC: Postabortion care
SAC: Safe abortion care
IV: Intravenous
PMA: Performance Monitoring for Action
SDP: Service devlivery point
ODK: Open Data Kit
LARC: Long-acting reversible contraception
GPS: Global positioning system
km: Kilometers
aOR: Adjusted odds ratio
EmOC: Emergency obstetric care
